# Dominance of Fructose-Associated *Fructobacillus* in the Gut Microbiome of Bumblebees (*Bombus terrestris*) Inhabiting Natural Forest Meadows

**DOI:** 10.3390/insects13010098

**Published:** 2022-01-15

**Authors:** Ronalds Krams, Dita Gudra, Sergejs Popovs, Jonathan Willow, Tatjana Krama, Maris Munkevics, Kaspars Megnis, Priit Jõers, Davids Fridmanis, Jorge Contreras Garduño, Indrikis A. Krams

**Affiliations:** 1 Chair of Plant Health, Estonian University of Life Sciences, 51006 Tartu, Estonia; ronalds.krams@student.emu.ee (R.K.); jonwillowtree@gmail.com (J.W.); tatjana.krama@emu.ee (T.K.); 2 Department of Biotechnology, Daugavpils University, 5401 Daugavpils, Latvia; sergey.p@email.com (S.P.); marismunkevics@gmail.com (M.M.); 3 Latvian Biomedical Research and Study Centre, 1067 Riga, Latvia; dita.gudra@biomed.lu.lv (D.G.); kaspars.megnis@biomed.lu.lv (K.M.); davids@biomed.lu.lv (D.F.); 4 Department of Zoology and Animal Ecology, Faculty of Biology, University of Latvia, 1004 Riga, Latvia; 5 Department of General and Microbial Biochemistry, University of Tartu, 51010 Tartu, Estonia; priit.joers@ut.ee; 6 Escuela Nacional de Estudios Superiores Unidad Morelia, Universidad Nacional Autónoma de México, Morelia 58190, Mexico; jcg@enesmorelia.unam.mx; 7 Institute of Ecology and Earth Sciences, University of Tartu, 51014 Tartu, Estonia

**Keywords:** bumblebees, *Fructobacillus*, gut microbiome, natural habitat, nectar, pollinators

## Abstract

**Simple Summary:**

A vast array of microorganisms colonize invertebrates and vertebrates. Most of these microbes reside in the digestive tract, where they constitute the intestinal (gut) microbiome. Some microbes are commensal, coexisting with their host without causing harm, while others can be mutualistic or pathogenic. Mutualistic microorganisms perform many health-related functions such as promoting digestion and acquisition of nutrients; hormone regulation; maintenance and control of the immune system; regulation of homeostasis and stress physiology of the body; insecticide resistance; production of certain vitamins; and providing protection against pathogenic microorganisms, parasites, and diseases. Bee-specific bacterial genera such as *Lactobacillus*, *Snodgrassella*, and *Gilliamella* dominate the gut communities of many bumblebees. This study confirmed *Lactobacillus*, *Snodgrassella*, and *Gilliamella* as dominant gut bacteria of the buff-tailed bumblebee *Bombus terrestris* in the agricultural landscape. However, we show that the guts of *B. terrestris* from natural forest habitats can be dominated by fructose-associated *Fructobacillus* spp. Our findings may have important implications for understanding the ecological role of bumblebees and the reasons for the decline of key pollinators.

**Abstract:**

Bumblebees are key pollinators in agricultural landscapes. However, little is known about how gut microbial communities respond to anthropogenic changes. We used commercially produced colonies of buff-tailed bumblebees (*Bombus terrestris*) placed in three habitats. Whole guts (midgut, hindgut, and rectum) of *B. terrestris* specimens were dissected from the body and analyzed using 16S phylogenetic community analysis. We observed significantly different bacterial community composition between the agricultural landscapes (apple orchards and oilseed rape (*Brassica napus*) fields) and forest meadows, whereas differences in gut communities between the orchards and oilseed rape fields were nonsignificant. Bee-specific bacterial genera such as *Lactobacillus*, *Snodgrassella*, and *Gilliamella* dominated gut communities of *B. terrestris* specimens. In contrast, the guts of *B. terrestris* from forest meadows were dominated by fructose-associated *Fructobacillus* spp. Bacterial communities of workers were the most diverse. At the same time, those of males and young queens were less diverse, possibly reflecting greater exposure to the colony’s inner environment compared to the environment outside the colony, as well as bumblebee age. Our results suggest that habitat quality, exposure to environmental microbes, nectar quality and accessibility, and land use significantly affect gut bacterial composition in *B. terrestris*.

## 1. Introduction

Microorganisms have a significant influence on their hosts’ behavioral responses, reproductive output, and development [1,2], and compositional changes in the microbiome have important consequences on host activity and fitness [3,4]. The diversity, stability, and composition of microbial communities in the gut are affected by a number of factors such as habitat, season, host density, intensity of intraspecific and interspecific competition, diet, exposure to agrochemicals and antimicrobials, and interactions between populations of the gut microbial community [2,3,4,5]. Studies on these factors and their interactions need more attention in order for us to further understand the role of the microbiome.

The ecological, physiological, and evolutionary functions of the microbiome are especially important to understand in keystone species [6]. These organisms have a disproportionately large effect on the ecosystems they inhabit. Many pollinators are keystone species because flowering plant communities could substantially change without their pollination services. A recent study indicates significant declines in insect populations, including a steep drop in pollinator numbers worldwide [7]. Pollination is critical for sustainable food production in human-managed ecosystems, as most flowering plant species only produce seeds if animal pollinators visit their flowers and provide pollination services [8]. Indeed, pollinators such as insects, birds, and bats affect almost 40% of global crop production [9].

In agroecosystems, pollinators such as bumblebees are essential for orchard, horticultural, and forage production, as well as production of seeds for many root, oil, and fiber crops [10,11,12]. Bumblebees are among the most effective pollinators for many wild plants in naturally occurring habitats such as forests and meadows [10]. It has been shown that resource acquisition in different types of habitats leads to changes in the composition and abundances within microbial gut communities in bumblebees, which is crucial for the bumblebees’ general condition and their resistance to pathogens [3]. Koch and Schmid-Hempel [13] found that bumblebee gut microbiomes usually consist of *Snodgrassella* spp. (Beta, Gammaproteobacteria, *Neisseriaceae*), *Gilliamella* spp. (Gamma-1 phylotype, Gammaproteobacteria, *Orbaceae*), *Lactobacillus* spp. (Firm-4/Lacto-2 and Firm-5/Lacto-1, Firmicutes, *Lactobacillaceae*), and *Bifidobacterium* spp. (Actinobacteria) [1,3,14,15,16,17]. A recent study showed the constant presence of yeasts across developmental phases [18]. Besides microbiome variations due to habitat, bumblebee microbiome composition depends on the social caste of an individual bumblebee. It was shown that core gut microbial communities can be different among castes of bumblebees [19]. Wang et al. [20] found that the genera *Gilliamella*, *Snodgrassella*, and *Lactobacillus* were relatively dominant in both unmated and ovipositing bumblebee queens, while *Bifidobacterium* spp. were dominant only in ovipositing queens. Adult worker bumblebees have a more specialized gut microbiota, dominated by *Gilliamella* spp., *Snodgrassella* spp., and *Lactobacillus* spp. [1,2,4,21,22]. These differences among queen, worker, and male microbiota stem from their diets during development or during the adult phase. For example, queen larvae are fed more royal jelly, and their diets are higher in protein, compared to the diets of future workers [23]. Pollen consumption and proteolytic activity in the gut are significantly lower in male honey bees than in both worker and nurse honey bees [24,25,26,27].

Associations between microbiota, habitat type, and social caste in insects suggest the role of diet and subsequent interactions with environmental bacteria [3]. Although microbiota may affect host-symbiont and host-pathogen dynamics, bumblebee colony health, and the quality of bumblebee pollination services in different habitats, interactions between habitat, caste, and microbiome are poorly understood. Therefore, we explored links between the microbiome and social caste in three habitats, using the model polylectic bumblebee species *Bombus terrestris* (L.) (Hymenoptera: Apidae), a widespread and abundant bumblebee species in Europe. In spring, we placed commercial *B. terrestris* hives in apple orchards, oilseed rape (*Brassica napus*) fields, and naturally occurring forest meadows. One month later, we collected workers, males, young queens, and ovipositing queens. Employing 16S rRNA amplicon sequencing, we investigated how the adult life stage and physiological state influence the gut microbiome among castes of *B. terrestris*. It has been recently shown that the microbiome of urban bumblebees is dominated by bee-specific core bacteria such as *Snodgrassella* spp. and *Gilliamella* spp., while the microbiome of *B. terrestris* in forests consisted of a number of small fractions of environmental bacteria [3]. Therefore, we predicted a higher diversity of bacterial species in bumblebees collected in the forest, compared to those collected in oilseed rape fields and apple orchards. We also predicted higher bacterial diversity, especially regarding environmental bacteria, in workers and ovipositing queens, since workers have many chances to obtain environmental bacteria during their everyday ecological interactions outside the hive, while ovipositing queens are the oldest individuals who probably had ample opportunity to contact the environment outside their hives.

## 2. Materials and Methods

### 2.1. Study Species, Collection of Specimens, and Bumblebee Habitats

Experiments were performed using commercial *B. terrestris* hives (Biobest Group NV, Westerlo, Belgium). At the beginning of May 2019, we placed hives in three different habitats in southeast Latvia: apple orchards, oilseed rape fields, and natural forest meadows. We had a total of 24 hives across eight apple orchards, 24 hives across eight oilseed rape fields, and 6 hives across forest meadows. Hives in apple orchards and oilseed rape fields were separated by at least 1.8 km. *B. terrestris* hives in forest meadows were placed at least 1.9 km apart. The meadows were located in Augšdaugava Nature Reserve, at least 2 km away from the nearest agricultural land or permanently inhabited rural property (55°52′46.95″ N, 27°14′4.65″ E). The forests had not been logged for 80–100 years, and the meadows had not been cut for 3–5 years. Specimens were collected at the beginning of July 2019, by collecting all hives from each site and placing the hives in a freezer (Angelantoni, Life Science, Milano, Italy) at −30 °C.

### 2.2. Gut Dissection and Microbial DNA Extraction

Prior to dissection, specimens were rinsed with 70% ethanol, pinned to a polyacrylamide gel plate, and immersed in sterile Ringer’s solution. The intestines were pulled out through the anus; the midgut and hindgut, including rectum, were collected into a vial with 1 mL of glycerol (40%) and homogenized using zirconia beads. Dissections were performed under a binocular microscope (Carl Zeiss AG, Jena, Germany). After each dissection, the Ringer’s solution was replaced and the gel plate was cleaned and disinfected with 70% ethanol. We pooled guts from three individual bumblebees, from all three hives from each study site, except for ovipositing queens whose samples contained only one individual per sample and male bumblebees from forest meadows whose samples were prepared from six individuals. In total, we had 93 samples from apple orchards, 93 samples from oilseed rape fields, and 24 samples from forest meadows (Table 1). Samples were prepared from a total of 540 *B. terrestris* individuals (Table 1). Microbial DNA from the dissected *B. terrestris* guts was extracted using FastDNA Spin Kit for Soil (MP Biomedicals, Santa Ana, CA, USA), according to the manufacturer’s guidelines. The concentration of extracted DNA was measured using a Qubit 2.0 Fluorometer and Qubit dsDNA HS Assay Kit (Life Technologies, Carlsbad, CA, USA).

### 2.3. 16S V3−V4 rRNA Gene Amplification and Illumina MiSeq Sequencing

Primers were designed for PCR amplification of the 16S rRNA V3−V4 region specific to the domain bacteria according to the 16S Metagenomic Sequencing Library Preparation protocol for Illumina MiSeq (Illumina Inc., San Diego, CA, USA). In brief, 4 ng of microbial DNA was amplified separately by V3 (341F) and V4 (805R) primers using Phusion U Multiplex PCR Master Mix (Thermo Fisher Scientific, Waltham, MA, USA) with the following reaction conditions: denaturation at 98 °C for 30 s; 35 cycles of 98 °C for 10 s, 67 °C for 15 s, and 72 °C for 15 s; and fragment elongation at 72 °C for 7 min. Yield of PCR products was assessed using 1.2% agarose gel electrophoresis and purified using NucleoMag NGS Clean-Up and Size Select kit (Macherey-Nagel GmbH & Co. KG, Düren, Germany). Concentration of the PCR product was measured using Qubit dsDNA HS Assay Kit on Qubit 2.0 Fluorometer (Thermo Fisher Scientific, Waltham, MA, USA). During the second stage of PCR, 4 ng of V3 and V4 PCR product was used to add Illumina MiSeq i7 and i5 indexes using custom-ordered Nextera XT Index Kit (Illumina Inc., San Diego, CA, USA) primers (Metabion International AG, Germany). For this reaction, Phusion U Multiplex PCR Master Mix was used with thermal cycler reaction conditions as specified above. The 16S rRNA PCR products were then pooled and purified for the sequencing reaction using NucleoMag magnetic beads. The quality and yield of 16S rRNA V3−V4 amplicons were assessed using Agilent High Sensitivity DNA kit on Agilent 2100 BioAnalyzer (Agilent Technologies, Santa Clara, CA, USA) and using Qubit dsDNA HS Assay Kit on Qubit 2.0 Fluorometer (Thermo Fisher Scientific, Waltham, MA, USA).

Before sequencing, all samples were diluted to 10 pM and pooled. Samples were paired-end sequenced using 500 cycles, using MiSeq Reagent Kit v2 on Illumina MiSeq. Each run was expected to produce at least 100,000 reads per sample. After the sequencing runs were completed, individual sequence reads were filtered using MiSeq software to remove low-quality sequences.

### 2.4. 16S Sequence Analyses

Sequence reads were de-multiplexed using Illumina’s MiSeq Reporter Software (Illumina Inc., San Diego, CA, USA) and quality filtered using Trimmomatic v.0.39 with the leading quality of Q20 and trailing quality of Q20, and sequences shorter than 36 bp were discarded. All quality-approved sequences were imported into the QIIME2 v.2019.1 environment for further analysis [28]. The DADA2 plugin was used to pair forward and reverse reads, as well as for extra sequence quality control and chimeric sequence removal using a pooled consensus method [29]. The resulting feature table and sequences were used for de novo clustering, employing the vsearch plug-in using a 97% identity threshold. Later, de novo multiple sequence alignment was performed using the MAFFT method [30], while phylogenetic trees were constructed using FastTree2 [31]. De novo clustered sequences were used for taxonomic assignment with a pre-fitted sklearn-based taxonomy classifier based on the Silva v.132 97% identity reference database trained with naïve Bayes classifier [32].

### 2.5. Statistical Analyses

Prior to statistical analysis, rarefaction was employed with the depth of 90% of the minimum sample depth in the dataset. Resulting depth per sample was 31 575 sequences. Alpha diversity (observed, Chao1, Shannon and Simpson indices) and beta diversity (weighted and unweighted UniFrac metrics) measures were calculated using the phyloseq v.1.30.0. package [33] in R v.3.6.3. [34]. Coverage for species richness was calculated by dividing the number of observed OTUs with Chao1 index measurement. Pairwise Wilcoxon rank-sum test with Holm’s *p*-value adjustment method was used to assess the significance of alpha diversity measurements between habitats and between castes. Permutational multivariate analysis of variance (PERMANOVA) was performed in QIIME2 to compare UniFrac distances between habitats. Next, the phyloseq package was used for network representation of bumblebees’ gut microbiomes. The network was built using the Bray–Curtis distance method with 0.7 as the maximum allowed distance between the two samples. In order to determine significant taxonomic entities across habitats and castes, differential expression analysis based on negative binomial distribution was performed using the DESeq2 v.1.26.0. package in R [35]. Taxonomic counts were normalized using log-relative transformation. All above-mentioned analyses were visualized using ggplot2 v.3.3.2. package [36].

## 3. Results

### 3.1. General Profile of the Sequencing Data

Illumina MiSeq sequencing of the bacterial 16S rRNA amplicons from different habitats and bumblebee castes yielded a total of 15,983,683 raw reads. After quality-filtering and read-merging, a total of 10,656,909 high-quality sequences remained. Based on 97% sequence similarity, after rarefaction 30,751 bacterial OTUs were obtained across all samples (Table 1). The total numbers of bacterial OTUs in the samples collected in apple orchards, oilseed rape fields, and forest meadows were 13,836, 12,210, and 4705, respectively. The total numbers of bacterial OTUs in the samples of males, young queens, ovipositing queens, and workers were 4185, 4811, 6006, and 15,749, respectively. The sample-based rarefaction curves of bacterial OTUs almost reached the saturation plateau (Figure S1). Average coverage regarding species richness for all samples was 74.45% ± 4.94% (Table 1), indicating that our sequencing depth was sufficient to cover the majority of bacterial taxa across habitat types and social castes.

### 3.2. Bacterial Community Composition

**Figure 1:**
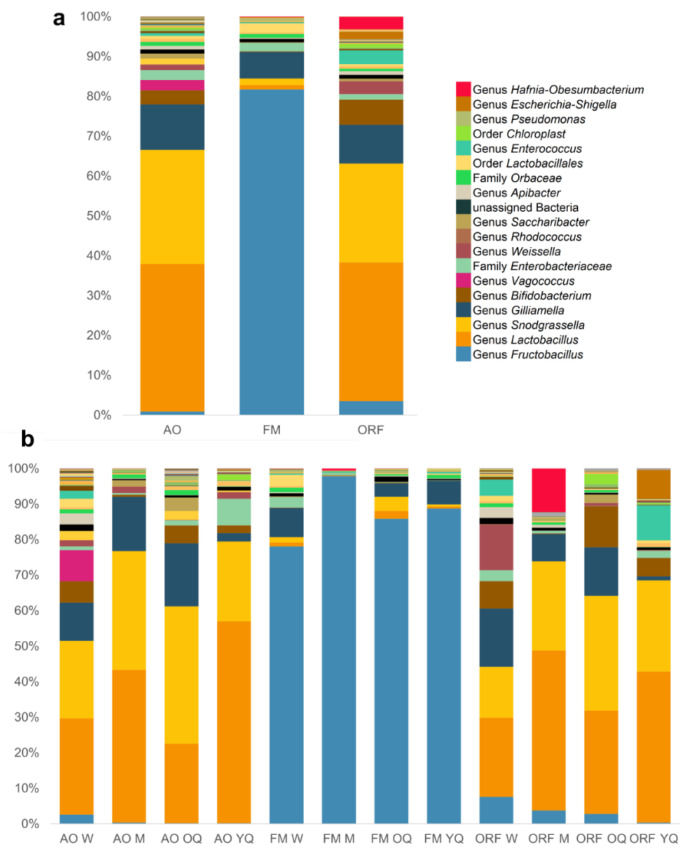
Relative abundance (%) of the bacterial genera found in the guts of bumblebee. (**a**) Distribution of bacterial genera within habitat; (**b**): distribution of bacterial genera for each caste within a habitat. AO: apple orchards, FM: forest meadows, ORF: oilseed rape fields, W: workers, M: males, OQ: ovipositing queens, YQ: young queens.

Bacterial OTUs in all samples were annotated into 12 phyla, 22 classes, 55 orders, 104 families, and 211 genera. The composition of bacterial genera among each habitat is shown in Figure 1a, with further subdivision among castes and sexes in Figure 1b at the phylum level. The gut microbiome of bumblebees mainly consisted of Firmicutes (52.7%), Proteobacteria (39.9%), Actinobacteria (5.2%), and Bacteroidetes (0.8%) (Figure 1a). At the genus level, the average relative abundance of bacteria belonging to *Lactobacillus*, *Snodgrassella*, and *Gilliamella* was over 1% across all habitats and castes. Among them, *Lactobacillus* was the most dominant (median 52.6% ± 132%, range 3.2–89.8%), followed by *Snodgrassella* (median 29.3% ± 183.8%, range 3.5–49.01%) and *Gilliamella* (median 12.3% ± 23.4%, range 0.02–39.95%).

**Figure 2:**
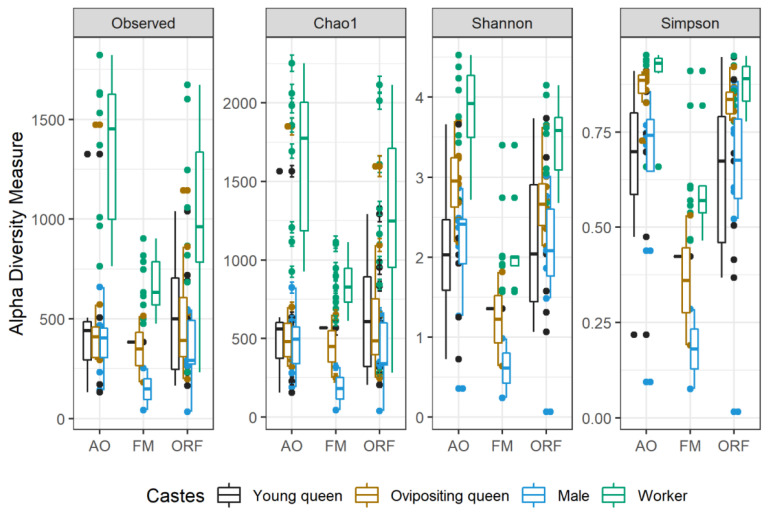
Alpha diversity indices (observed OTUs, Chao1, Shannon and Simpson diversity indices) of bumblebee castes in apple orchards (AO), forest meadows (FM), and oilseed rape fields (ORF).

Observed OTU richness varied between 99 and 1237 bacterial OTUs (mean of 446.3 OTUs) per sample in apple orchards, 26 and 1016 bacterial OTUs (mean of 393.87 OTUs) per sample in oilseed rape fields, and 31 and 598 OTUs (mean 336.07) per sample in forest meadows (Table 1). Gut microbial communities were more evenly distributed for bumblebee workers: in apple orchards, average Simpson’s index was 0.9 ± 0.09; in oilseed rape fields, average Simpson’s index was 0.88 ± 0.0; and in forest meadows, average Simpson’s index was 0.62 ± 0.15 (Figure 2).

### 3.3. Comparisons of Microbial Communities from Different Habitats and Castes

Richness and diversity of microbial communities across habitats and castes were estimated using alpha diversity indices, including observed richness (units in OTUs), Chao1 (units in OTUs), Shannon (index value), and Simpson (index value) estimators (Figure 2). Observed richness (*p* = 1) and Chao1 (*p* = 1) did not show significant differences between alpha diversity across habitats. In contrast, the Shannon diversity index showed significantly higher alpha diversity in apple orchard and oilseed rape field samples compared to forest meadow samples (*p* = 0.0016 and *p* = 0.0025, respectively). The Simpson index showed significantly higher diversity evenness in apple orchard and oilseed rape field samples compared to forest meadow samples (*p* = 0.0007 and *p* = 0.0015, respectively). Among castes, the number of observed OTUs was found to be highest in workers, and their observed alpha diversity significantly differed from that in males (Wilcoxon rank-sum test: *p* < 0.0001), young queens (*p* = 0.0007), and ovipositing queens (*p* = 0.0006; Figure 2). Other comparisons were not significant (*p* > 0.05; Figure 2). Chao1 showed similar results: alpha diversity richness was the highest in workers, and their observed alpha diversity significantly differed from that found in males (Wilcoxon rank-sum test: *p* < 0.0001), young queens (*p* = 0.00034), and ovipositing queens (*p* = 0.00021; Figure 2). Shannon diversity index showed significant differences in alpha diversity between workers and males (Wilcoxon rank-sum test: *p* = 0.003), between ovipositing queens and males (*p* = 0.028), and between workers and young queens (*p* = 0.028). All other comparisons did not show significant differences (*p* > 0.05). The Simpson index differed significantly between males and ovipositing queens (*p* = 0.0098) and between males and workers (*p* = 0.024).

**Figure 3:**
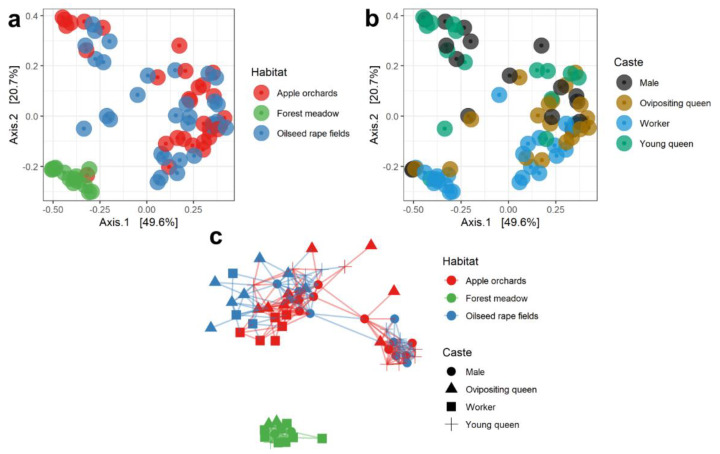
Metric multidimensional scaling (MDS/PCoA) on weighted UniFrac distances of the gut bacterial community from *Bombus terrestris* specimens collected from three habitat types (**a**) and from four castes (**b**). (**c**) Network analysis of *B. terrestris* gut bacterial community clustering by habitat, based on the Bray–Curtis dissimilarity matrix. Distance between different points on the plot reflects similarity level: the more similar the communities, the smaller the distance between the points.

Microbial community structure comparisons between different habitats were performed using multidimensional scaling (MDS) based on UniFrac distances and showed clear separation of gut bacterial community composition associated with forest meadows (Figure 3a). Network analysis based on the Bray–Curtis dissimilarity matrix supported this observation (Figure 3c). PERMANOVA analysis showed significant differences in bacterial community MDS distances of bumblebees sampled from the three habitat types. Beta diversity distances were significant between apple orchards and forest meadows (*n* = 45, pseudo-F = 7.47, *p* = 0.001 for unweighted UniFrac; *n* = 45, pseudo-F = 23.89, *p* = 0.001 for weighted UniFrac) and between forest meadows and oilseed rape fields (*n* = 45, pseudo-F = 6.32, *p* = 0.001 for unweighted UniFrac; *n* = 45, pseudo-F = 15,035, *p* = 0.001 for weighted UniFrac). Network analysis of the gut bacterial community, based on Bray–Curtis dissimilarity matrix, showed even clearer differences between bacterial communities of bumblebees collected from apple orchards, oilseed rape fields, and forest meadows (Figure 3c).

**Figure 4:**
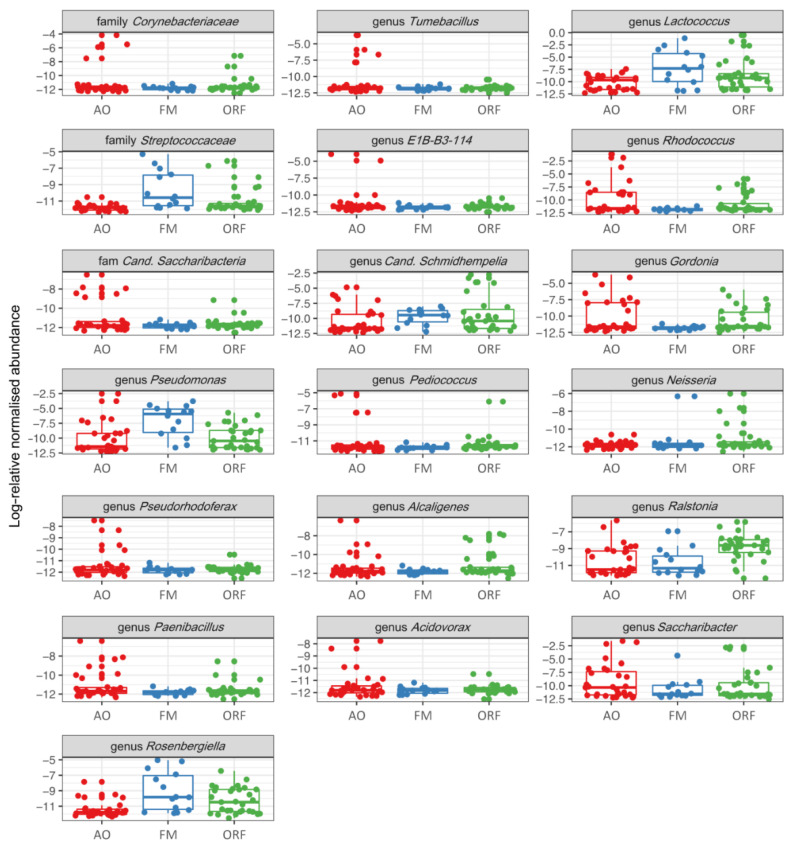
Log-relative abundance of the differentially expressed bacterial genera in the guts of *Bombus terrestris* collected from apple orchards (AO), forest meadows (FM), and oilseed rape fields (ORF).

**Figure 5:**
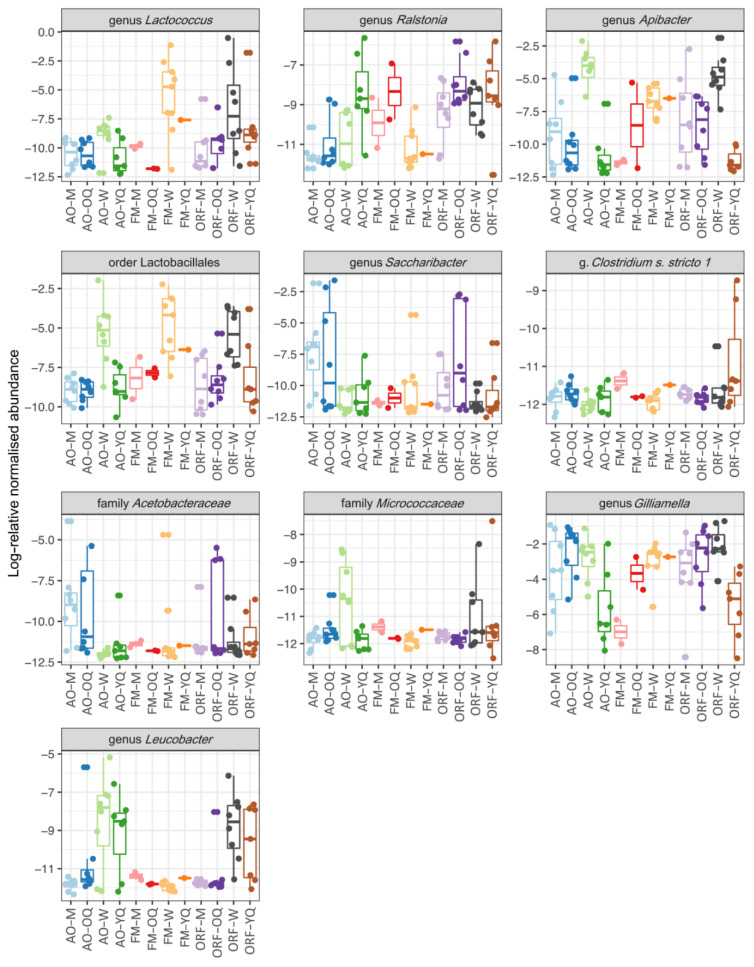
Log-relative abundance of the differentially expressed bacterial genus in the guts of *Bombus terrestris* collected from apple orchards (AO), forest meadows (FM), and oilseed rape fields (ORF), as well as across males (M), ovipositing queens (OQ), workers (W), and young queens (YQ).

The core bacterial genus *Lactobacillus* showed the highest relative abundance in the guts of *B. terrestris* specimens. These bacteria were more abundant in specimens collected from apple orchards (36.9%) and oilseed rape fields (33.3%) compared to forest meadows (1%) (Figure 1a and Figure 4). These bacteria were found in all castes’ microbiome, and castes did not differ with regard to abundance of *Lactobacillus* spp. (*p* > 0.05). The second most abundant core bacterial genus, *Snodgrassella*, was also more abundant in specimens collected from oilseed rape fields (23.8%) and apple orchards (28.5%) compared to forest meadows (1.5%). The presence of *Snodgrassella* spp. occurred across all castes. *Gilliamella* spp. were found in specimens collected from all three habitats, and these bacteria were less typical for young queens. In forest meadows, the most abundant bacteria were *Fructobacillus* spp. (74.01%). These bacteria were also found in all other habitats and castes, but their abundance in oilseed rape fields (3.39%) and apple orchards (0.93%) was significantly lower (*p* < 0.0001)*. Fructobacillus* spp. were less typical in the microbiome of young queens collected from apple orchards and oilseed rape fields. *Bifidobacterium* spp. were mostly found in apple orchards and oilseed rape fields; this genus was typical for workers, ovipositing queens, and young queens and less typical for males, which differed in their microbiome from all other castes (Figure 3b and Figure 5).

Less common bacterial taxa detected in the guts of *B. terrestris* specimens collected from apple orchards included *Apibacter* (1%), *Orbaceae* (1%), *Saccharibacter* (1.3%), *Rhodococcus* (1.4%), *Weisella* (1.4%), *Enterobacteriaceae* (2.5%), and *Vagococcus* (2.6%). Less common bacterial taxa detected in the guts of *B. terrestris* specimens collected from oilseed rape fields included *Lactococcus* (3.7%), *Hafnia-Obesumbacterium* (3%), *Escherichia-Shigella* (1.8%), *Enterococcus* (3.3%), *Weisella* (3.1%), *Enterobacteriaceae* (1.3%), and *Orbaceae* (0.6%). *Lactococcus* (3.6%), *Staphylococcus* (0.03%), *Pantoea* (1.9%), *Lactobacillus* (1%), *Orbaceae* (0.9%), and *Enterobacteriaceae* (1.9%) comprised a minor part of the microbiome of bumblebees collected from forest meadows.

## 4. Discussion

We found that bacterial community composition significantly differed between *B. terrestris* specimens collected from forest meadows and specimens collected from agricultural landscapes. This is in contrast to results from other studies [13,15,22], which observed that the composition and richness of OTUs in the bumblebee gut microbiome differs between species while remaining consistent over different locations and habitats. Regarding *B. terrestris*, previous studies have attributed this consistency to endosymbiont-specificity, including host-specific specialization of bacteria from the genera *Gilliamella* and *Snodgrassella* [22,37]. In forest meadows, habitat with the least amount of anthropogenic impact, the microbiome of *B. terrestris* was dominated by the genus *Fructobacillus*, whereas the genera *Lactobacillus*, *Snodgrassella,* and *Gilliamella* were the core bacterial community members in specimens collected from oilseed rape fields and apple orchards.

Members of the genus *Fructobacillus* are classified as fructophilic lactic acid bacteria [38]. These bacteria require fructose for carbohydrate catabolism and are therefore found in fructose-rich environments, such as fruits and nectar-rich flowers [39]. *Fructobacillus* spp. have been shown to be represented in the larval gut community in honeybees [40], as well as in adult honeybees [13,41,42] and adult bumblebees [22]. The necessity for fructose is due to defective alcohol fermentation in these bacteria, caused by the absence of the alcohol/acetaldehyde dehydrogenase gene *adhE* [43]. This inhibits reoxidation of NADH, via fermentation back to the electron acceptor NAD^+^, which is an essential cofactor for glycolysis function. To rebalance the NAD^+^/NADH ratio, these bacteria use other compounds as electron acceptors, including pyruvate, O_2_, fructose, and phenolic compounds such as coumaric acid [44]. Therefore, supplementing the growth environment with pyruvate, fructose, and aerobic conditions can enhance capacity for glycolysis and greatly improve bacterial catabolism of glucose [45]. Thus, in addition to being a carbon source, fructose facilitates the consumption of different carbohydrates via glycolysis.

There are several explanations of why *Fructobacillus* spp. are underrepresented in agricultural habitats. Apple orchards provide pollinators with both pollen and nectar, yet the blooming season of apple trees is short, and bumblebees occurring in these habitats rely on apple flowers only during one to two weeks and then must locate other sources of nectar and pollen. This may explain the high observed diversity in the gut microbiome of *B. terrestris* specimens collected from apple orchards. In addition, while the blooming season of oilseed rape is longer than that of apple trees, oilseed rape nectar and pollen are available within a relatively short period compared to nectar and pollen availability in forest meadow flowering plant communities. *B. terrestris* is a relatively large insect pollinator that uses floral characteristics such as flower size [46] as visual signals of resource availability, while flowers of oilseed rape are smaller and potentially less attractive to bumblebees, compared to honeybees, and flower size may represent an indicator of nectar production [47,48]. Moreover, oilseed rape typically produces nectar with high glucose content, resulting in rapidly crystallizing honey [49]. This kind of nectar cannot sustain *Fructobacillus* bacteria in the gut, because the use of glucose as a carbon source in *Fructobacillus* spp. is limited. Cold stress also causes physiological and biochemical changes in plants, which modifies sugar levels in nectar [50]. It has been shown that spring frosts increase fructose levels in nectar [51]. It has also been shown that raising ambient temperatures decreases the amount of sucrose, glucose, and fructose in nectar, as the secretion of these compounds declines in response to stressors such as climate change [52,53]. Finally, flowering plants must compete for pollinators in areas with high densities of native flowering plants, often leading to higher sugar content in nectar [54]. These factors may increase fructose concentration in nectar and provide optimal conditions for permanent occurrence of *Fructobacillus* spp. as dominant microbes in the guts of *B. terrestris*, especially close to the northernmost range of the distribution of *B. terrestris* [55,56,57]. The dominance of *Fructobacillus* spp. may be one reason for lower representation of *Gilliamella*, *Snodgrassella*, *Bifidobacterium,* and other genera in the guts of *B. terrestris* specimens collected from forest meadows in the present study.

Fructophilic lactic acid bacteria produce antimicrobial hydrolases, form biofilm that suppresses growth and formation of harmful microorganisms (e.g., *Pseudomonas aeruginosa*), consume toxic saccharides formed by catabolism of pollen, and catabolize phenolic acids into more potent antioxidants [58]. Their beneficial effects on resistance to pathogens have been confirmed in honeybees and the greater wax moth (*Galleria mellonella*) [59,60,61]. Our observed striking differences in presence of *Fructobacillus* spp. in *B. terrestris* microbiomes, between natural habitats and agricultural habitats, suggest that bumblebees feeding in agricultural areas may be more susceptible to pathogens and parasites [62] and less resistant to changes in the environment. *Fructobacillus* bacteria are highly susceptible to antibiotics [63], and if beekeepers use antibiotics to treat bacterial diseases of honeybees, this can lead to the depletion of *Fructobacillus* bacteria in the honeybee gut [63]. If honeybees in the agricultural environment leave antibiotic residues on flowers, bumblebees may collect these residues together with nectar and pollen of contaminated flowers, reducing the amount of *Fructobacillus* spp. in the gut of bumblebees. This suggests potentially harmful effects of increasing agricultural area on bumblebee population fitness, potentially resulting in decreasing numbers of this important pollinator species, even if abundant floral resources are present. Overall, our results support a recent study showing that the higher species biodiversity and abundance of bumblebees are positively associated with the proportion of forests in the landscape [64]. Our results also suggest that bumblebees might be more resistant to pathogens in wooded areas. This needs to be tested in the future by sequencing 16S rRNA region from bacteria and ITS2 region from fungi [65].

When bumblebees transport food to their nest, the food is often passed from one individual to another via trophallaxis, a food exchange mechanism. *Lactobacillus* is a bacterial genus commonly associated with pollen, and it may be spread among colony members or obtained when consuming stored food [56,66]. In the present study, workers had overall the most diverse bacterial composition within the gut, which may be explained by their exposure to other bumblebees, the nest environment, and the environment outside the nest. Exposure to the nest environment, feces, and food and interactions with other nest members are essential for colonization by Gram-negative core bacterial genera such as *Gilliamella* [57]. As the youngest individuals, young queens had the shortest time of exposure to the nest environment, possibly explaining the smaller relative abundance of *Gilliamella* spp. in *B. terrestris* specimens collected from apple orchards and oilseed rape fields. Finally, the lower OTU diversity in the gut microbiome of male *B. terrestris* specimens collected from forest meadows may be explained by their shorter exposure period to the environment outside the nest.

To our knowledge, our submission represents the first study to demonstrate the dominant representation of the bacterial genus *Fructobacillus* within the microbiome of any pollinator species. Although some previous studies found *Fructobacillus* to be a common bacterium in the gut of bumblebees [13,67,68], *Fructobacillus* has never been shown to be the most dominant microorganism in the gut of pollinators. As the prevalence of this genus was here demonstrated in a model bumblebee species, and in specimens associated with natural forest meadow habitat, our study has clear implications for conservation of natural habitats, wild pollinator nutrition, and fitness at both the individual and population scale. This is important because forest vegetation in northern Europe has evolved in the presence of large wild or domestic grazers [69]. This type of meadow has high plant and insect species richness, where plant species densities reach the highest values in the world. For example, the number of vascular plants in some Estonian wooded meadows can be as high as 76 plant species per m^2^ [70]. Since the existing pollinator communities could have evolved when wooded meadows dominated the agricultural ecosystems, it would be essential to study bumblebee communities and the microbiome of bumblebees in the remaining wooded meadows and in the areas where wooded meadows are actively restored. This would be important to study links between habitat, diversity of environmental bacteria, bumblebee social organization, bumblebee microbiome, and the role of bumblebees in local ecosystems.

## 5. Conclusions

We collected *B. terrestris* specimens from natural forest meadow habitat and two agricultural landscape types and characterized the bacterial community composition present in the guts of collected specimens. Our results showed dominance of *Lactobacillus* spp. and *Snodgrassella* spp. in the guts of bees collected from both apple orchards and oilseed rape fields. In contrast, *Fructobacillus* spp. were dominant in the guts of bees collected from unmanaged forest meadows. The low prevalence of *Fructobacillus* spp. in guts of *B. terrestris* specimens collected from both apple orchards and oilseed rape fields, compared to their more natural reference habitat, could suggest a potential stressor to *B. terrestris* populations in agricultural landscapes [71]. This low prevalence may be alleviated via the preservation and/or restoration of nectar-rich forest meadow habitat within agricultural landscapes. These conserved habitats should contain flowering plant communities that provide suitable floral resources over longer durations than those observed in crop-dominated habitats.

## Figures and Tables

**Table 1:** Mean diversity measures (±standard deviation) of gut bacterial communities in *Bombus terrestris*.

Habitat Type	Caste	Number of Insects Used (Number of Samples)	Observed OTU Richness	Chao1	Coverage (%)	Shannon	Simpson
Apple orchards	ovipositing queen	24 (24)	356.5 ± 239.48	468.83 ± 352.81	74.2 ± 4.91	2.92 ± 0.48	0.86 ± 0.06
	young queen	63 (21)	311 ± 227.07	428.3 ± 323.22	73.37 ± 7.81	2.07 ± 0.96	0.66 ± 0.24
	worker	72 (24)	841.75 ± 253.51	1156.98 ± 352.39	72.9 ± 3.31	3.82 ± 0.59	0.89 ± 0.09
	male	72 (24)	259.13 ± 90.78	345.63 ± 137.85	75.89 ± 6.07	2.05 ± 0.83	0.65 ± 0.26
Oilseed rape fields	ovipositing queen	24 (24)	336.13 ± 191.18	450.09 ± 269.67	76.22 ± 4.28	2.71 ± 0.46	0.83 ± 0.05
	young queen	63 (21)	338.57 ± 177.04	455.59 ± 231.6	74.25 ± 5.69	2.21 ± 1.01	0.64 ± 0.23
	worker	72 (24)	659 ± 264.68	895.48 ± 376.07	74.58 ± 5.03	3.46 ± 0.49	0.88 ± 0.06
	male	72 (24)	234.88 ± 114.47	308.42 ± 146.96	76.17 ± 3.01	1.98 ± 0.92	0.62 ± 0.28
Forest meadows	ovipositing queen	6 (6)	232.5 ± 196.66	288.1 ± 196.66	81.06 ±1.1	1.22 ± 0.82	0.36 ± 0.24
	young queen	18 (6)	264	372.78	70.82	1.36	0.429
	worker	18 (6)	415.89 ± 93.36	583.84 ± 131.11	71.32 ± 2.2	2.12 ± 0.59	0.62 ± 0.15
	male	36 (6)	116.5 ± 120.92	173.5 ± 188.79	71.65 ± 8.28	0.61 ± 0.52	0.18 ± 0.15

## Data Availability

Raw sequence data have been deposited at the European Nucleotide Archive under study accession No. PRJEB48765. Taxonomical summaries have been deposited at the Zenodo repository (doi: 10.5281/zenodo.4677288).

## Supplementary Materials

The following supporting information can be downloaded at: https://www.mdpi.com/article/10.3390/insects13010098/s1, Figure S1: Rarefaction curves of bacterial OTUs and relative abundance of bacteria. Rarefaction curves show the number of gut bacterial OTUs associated with each habitat type. Rarefaction curves reached saturation, suggesting that the sequencing depth covered the most abundant bacterial community members.
