# Supplementary material for: Dominance of Fructose-Associated Fructobacillus in the Gut Microbiome of Bumblebees (Bombus terrestris) Inhabiting Natural Forest Meadows
Source: Insects. 2022 Jan 15;13(1):98. doi: 10.3390/insects13010098 (PMC8779478; doi:10.3390/insects13010098)
Supplement: Supplementary file 1 [file insects-13-00098-s001.zip › insects-1506089-supplementary.pdf]

Supplementary figure

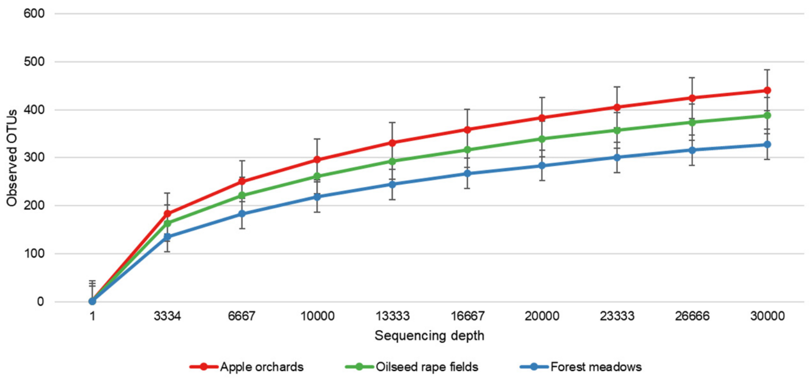

Figure S1. Rarefaction curves of bacterial OTUs and relative abundance of bacteria. Rarefaction curves show the number of gut bacterial OTUs associated with each habitat type. Rarefaction curves reached saturation, suggesting that the sequencing depth covered the most abundant bacterial community members.
